# Collaborative Experience Success Stories in Integrated Care of Older People: A Narrative Analysis

**DOI:** 10.5334/ijic.5452

**Published:** 2020-08-17

**Authors:** Inger Kjellberg

**Affiliations:** 1 Department of Social work, University of Gothenburg, Sprängkullsgatan, Göteborg, SE

**Keywords:** integrated care, collaboration, elderly care, storytelling, narrative

## Abstract

**Introduction:**

Inter-organisational collaboration is crucial in the care of older people, as is the development of integrated care. Storytelling in organisations is one way of understanding how to achieve successful collaboration. This article provides insights into the ways in which storytelling in collaborative experiences contributes to a collective identity instrumental in the successful collaborations involved in integrated care for older people.

**Theory:**

Managing cultural diversity is one specific theme in the theory of collaborative advantage; this is used in combination with theories of storytelling in organisations.

**Method:**

Interviews with staff from three different municipalities applying three various strategies for integrated care were carried out. Stories of the collaborative experiences were analysed using a narrative approach.

**Results:**

The most significant finding was that a similar type of success story was evident across all three municipalities. The story was identified as an epic-comedy story where success was accomplished through the heroic characterisations of the managers, in addition to their improvisation abilities and discretionary work towards common goals.

**Conclusion:**

It is suggested that storytelling in collaborative experiences is one way of overcoming cultural frictions between different collaborating actors and may contribute to a coherent sense of a collective identity, thus facilitating further collaboration.

## Introduction

The call for integrated care has been induced by demographic change, increased fragmentation of the welfare sector and increased demand for technically and more expensive care solutions [1,2]. There is a wide variety of strategies and initiatives to improve care continuity and coordination for people with chronic diseases, not least for older people with complex care needs who require care from multiple providers [3]. Many factors have been emphasised as beneficial for the success of joint working and integration, for instance co-location, team building events and pooled budgets [2,4].

In Sweden, many local programmes and projects aim to advance collaboration and integrated care in the health and social care of older people. Inter-organisational collaboration is crucial due to a division in the care sector in Sweden, where the counties govern healthcare and the municipalities are responsible for social care. In order to achieve integrated care, inter-organisational collaboration can generate collaborative advantages through the amalgamation of different partners’ resources and expertise [5]. However, many collaborations fail and result in collaborative inertia instead of advantages due to barriers such as organisational differences, lack of agreement of responsibilities, and different communication and information systems [6]. Differing organisational and professional cultures are factors often raised as barriers to successful integrated care [4,5,7].

Culture, in this context, refers to “habitual ways of being and acting that stem from the distinct professional, organisational, and national cultures to which they belong” [8, p. 59]. Organisational culture is also broadly defined as patterns of shared assumptions learned in a group, which are subsequently taught to new members [9]; these are maintained by the stories and myths told and retold both by members of the organisations and outsiders [10]. Frequently telling stories has also been suggested to help establish organisational identity [11], and the importance of coherent narratives for organisations in regard to both their legitimacy and very existence has been underlined [12,13]. Along the same vein as this branch of narrative organisational research, the stories told by members in collaborating organisations in integrated care is yet another way of understanding the paths to successful collaborations between health and social care.

In this article, the stories told about collaborating experiences in health and social care for frail older people are analysed and understood as key to the development of integrated care. A growing body of research [11,12,14] suggests that an analysis of such stories may underpin a much needed understanding of organisational life. In integrated care, different organisations must collaborate and coordinate their services. Consequently, this study focuses on storytelling in three different projects aiming to provide integrated care for older people in Sweden by exploring the type of stories and the function of storytelling for a successful collaboration. It is the similarities between the stories in the three municipalities engaged in various strategies to integrated care that are the focus of the analysis, and it builds on interviews with staff from different levels in health and social care. The contribution to research on integrated care is to provide insights into the ways in which storytelling in collaborative experience contributes to a collective identity instrumental in the successful collaboration and integration of care for older people.

## Theoretical framework

The theory of collaborative advantage is practice-based and concerns the management of collaborations structured in overlapping themes causing anxiety or reward [6]. The specific theme examined here is the tensions in managing cultural diversity, which are analysed using storytelling in the collaborative organisations. The organisations have different structures and procedures and when distinct resources can be deployed jointly, collaborative goals can be pursued. This requires flexibility; each organisation has their own internal purposes, and these may have to be adjusted during the process of collaboration. Paradoxically, such flexibility may comprise an organisation’s core contribution to the collaboration; too many adjustments to collaborating partners can hinder the deliverance of their specific part, resulting in tensions between flexibility and rigidity [8]. Cultural frictions may also arise from differing expectations of what can be achieved within the collaborative context, resulting in tensions between autonomy and accountability. Managers need individual autonomy to act on behalf of their organisations but they also need to protect and maintain its accountability [8].

Stories are sense-making devices that help us to process our experiences, communicate with others and aid comprehension by conveying shared values [15]. Stories have also been noted for effectively developing organisational or group identities [16]. Organisations consist of people, and an organisation’s identity is characterised by multiple narratives and shared storylines due to negotiations and networking; the summation of these shared stories surface as a collective identity [17]. The terms ‘narrative’ and ‘story’ are often used interchangeably and both include a chronological order and a thematic ordering of events [14]. Both narratives and stories need a plot which ties together the parts in a meaningful whole, and narration has been recognised as the process in which narratives are told, whereas storytelling is the activity that spreads different kinds of stories in and around organisations [14]. In this article, no further distinction between the terms narrative and story is made, but the latter is used as storytelling [10] is the focus of the analysis. Gabriel [10] distinguishes between four main types of stories: epic, comic, tragic and romantic. Different combinations of these types lead to hybrids, for example epic-comic stories. Epic stories deal with achievements and contests, and most result in a happy ending. Comic stories generate laughter and amusement through themes such as mishaps and misfortune with a comical twist. In contrast, tragic stories include traumas and insults emerging from injustice and unfairness with emotional responses like anger or fear, and romantic stories engage with love, gratitude and affection. In epic stories, the protagonist is a hero often waging against bureaucracy and the resolution is reached by courage and force. An interesting hybrid is the epic-comic type of story; a courageous hero fighting against a bureaucratic system with a mixture of improvisatory qualities, wit and cunning [10]. Hibbert and Beech [18] suggest that there are recognisable characterisations commonly appearing in collaborative stories such as heroes and villains; however, the occupants of the principal roles may change and are not assumed to be constant. Success stories in, for example, post-merger integrations have also been investigated [19], illustrating that these often hold a linear logic of successive temporal events underlining the actions of the top management as the causes of success leading to overly optimistic views on the managements’ ability.

## Method

### Setting

Sweden comprises 21 counties and 290 municipalities. The counties govern the healthcare delivery, including hospitals and specialised and primary care. The municipalities are responsible for the social care of the elderly, including home care, special housing (nursing homes) and caregiver support. In almost all counties, the municipalities are also responsible for home healthcare. Multi-professional teams for older people are hospital-based, although they collaborate with nurses in the municipal home healthcare and with home care facilities. Thus, integrated care for older people in Sweden involves horizontal coordination between the county and municipal authorities and inter-organisational collaboration between providers of municipal social care and county healthcare agencies, as well as interprofessional collaboration. Such collaboration mainly takes place in multi-professional teams and in the interface between home healthcare and multi-professional teams, and physicians in primary care and professionals in specialised care and rehabilitation.

The findings reported herein were part of a larger research project addressing collaboration between health and care services for older persons with complex needs. The research was conducted across three municipalities in the south of Sweden. These municipalities were strategically selected to include a variety of integrated care strategies where there was evidence of good practice. Good practice for older people in Swedish municipalities was sought after in two ways: the specific indicators for the coordinated care of older persons in a bench-marking system, called open comparisons [see, for example, 20] and evaluations [21,22]. In the municipalities, three differing strategies for integrated care were deployed. The first municipality (A) used an acclaimed network care model known as the “Esther-project”, with an explicit focus on what was best for the older person, symbolically called ‘Esther’. The model builds on collaboration between health and care organisations on a voluntary and equal basis without hierarchical structures [see 23]. The main strategy for integrated care in the second municipality (B) was the unique pooling of resources manifested in one interim ward, which was equally used by both the county and the municipality for patients not quite ready to leave hospital or for service users with home care in need of a few days of medical care and supervision. The interim ward was situated at the primary care clinic and was staffed by registered nurses around the clock and physicians during the daytime and on call during nightshifts. The costs for the ward were shared between the municipality and the county. In the third community (C), the main strategy for integrated care was multi-professional teams (the ‘Skaraborgs’ model) [24]; two different teams (geriatric and palliative) collaborated with one primary care physician who was appointed to work with the home healthcare nurses and with home care in the municipality. The team-based care initiative was part of a larger, ongoing collaboration between health and social care in several municipalities. Although overall the three municipalities had different strategies for integrated care, they were all good examples of well-functioning collaboration in the care of older people. The reason for including them in the project was to identify whether there were common structures for successful collaborations, although the strategies for integrated care differed.

In all three municipalities, the population was approximately the same; ranging from 15 952 to 21 758 inhabitants, with a slightly higher population of older people (18 percent) than in Sweden (15 percent) in general [25]. The municipalities were located in three different counties (see Table 1).

**Table 1:** Participating municipalities, integrated care approach, number of interviews and interviewees’ roles.

	Municipality A	Municipality B	Municipality C

Population (2018)*	17 677	21 758	15 952
County	Jönköping	Östergötland	Västra Götaland
IC approach	Network	Pooling resources	Multi-professional teams
Name of project	Esther	Interim ward	Skaraborgs model
Interviews (no of respondents)	6(13)	11(13)	6(6)
Role of interviewees: Managerial level	social services, home healthcare, home care, hospital coordinator	social services, home healthcare, primary care unit	social services, home healthcare, home care, team coordinator
Staff	nurse aides, caregiver support	physiotherapists, reg. nurses, physicians, caregiver support	caregiver support

* [25].

### Material

Interviews (n = 23) with staff from healthcare and social care in the three municipalities were carried out; these included interviews with the head managers of the municipal social services, as well as managers and staff from home healthcare, home care and nursing homes, caregiver support, physicians and paramedics from primary care units, staff from multiple teams and the coordinators of care teams. The interviews took place in the interviewees’ workplaces in spring 2017, and each interview lasted from between 40 minutes to 2 hours. Most interviews were individual (18), although five were group interviews (2–4 respondents). The interviews were semi-structured and the interview guide contained two broad themes: 1) the organisation of health and social care for frail and older people in the municipality from the respondent’s point of view; and 2) challenges and successes with the coordination of care for the target group in the municipality. The themes in the interview guide were influenced by the overall purpose of the research project; to describe and analyse differences in local collaboration in health and care services for older people with complex needs. Altogether, 32 respondents were interviewed; 13 people were interviewed from both municipals A and B, and six people in municipal C. More staff were asked to participate but declined, mostly due to a heavy workload. All interviews were recorded and transcribed verbatim. Participant validation was carried out by writing a short summary of the main findings, administered to the head manager of care and social services and the interviewees. They were invited to comment on the interpretation of their care model and their interviews. The feed-back from the interviewees concerned a few misunderstandings of factual statements such as how many people the home health care was caring for and details about the multidisciplinary teams.

The integrated care projects had been in use for over twenty years in municipality A, for fourteen years in municipality B, and for eighteen years in municipality C. Some of the interviewed managers and coordinators had been personally involved in the development of each municipality’s strategy of integrated care since its beginning, telling stories of how it all began, while some of the staff were more interested in telling stories of care work on a day-to-day basis, which included more practical problems and conflicting perspectives. More interviews with respondents from a managerial level were carried out in municipalities B and C, whereas there were more staff from home care, nursing homes and social services who worked in closer contact with service users in municipality A. If more staff, in contrast to managers, had been interviewed in municipalities B and C, the chance of hearing more contradictory stories may have been greater.

### Analysis

Initially, a thematic analysis [26] was conducted, and a coding frame was inductively generated from the interviews in one municipality. The coding frame was then used and elaborated on in the next two municipalities. The final themes from this analysis included: collaboration in practice, the key goal of integrated care, organisational structures, the “small” municipality, common goals, advantages of collaboration, challenges, trust, self-presentations and “the story of collaboration”. The last theme, “the story of collaboration” included the interviewees’ stories of how their work with the specific integrated care strategy had developed, and this theme is the material for the narrative analysis in this article. The narrative analysis proceeded in two steps. Following Gabriel [10], the focus was on complete stories with a beginning, middle and end. First, the chronological order of the interviewees’ stories of the development of the integrated care project was carried out. These stories were mainly told by the managers who had been involved in the change, but other participants also repeated it. Then the type of story was identified, and the plot and characters were compared, in line with Gabriel’s characteristics. According to Gabriel [10, p 83], each type of story represents a distinct poetic mode, a particular way of “turning facts into meaningful, emotionally charged stories”, and the stories have different plot focus, predicament, poetic tropes and emotions. For example, in an epic-comic story, the hero has humour, other characters are villains, victims and accomplices. The plot focus is a display of wit and unorthodox achievements. This type of story evokes admiration and mirth in comparison with an epic-tragic story which would give rise to amusement, but also pity, fear and guilt. In the second step, all transcripts were scrutinised carefully again, in search of sequences or conflicting stories due to cultural frictions between different levels of staff and organisations [8]. In particular, individual and collective self-presentations, as claims for success was noted, directing the analytical attention on collective identity as a discursive construct [17]. At this stage, other stories was found, for example of how staff managed difficult care situations. However engaging in their own right, these stories were excluded as they did not focus on collaborative experiences. A critical reflection on Gabriel’s focus on whole stories with the intention of entertain and evoke emotions [10] is that such a narrow view on stories may restrict the analysis. Nevertheless, in the interviews, there was a very distinct story about collaboration in each integrated care project. It was as if the story of how it all started, the charismatic leaders of the project and their struggle to overcome challenges and hardships had turned into a collective tale re-told by the staff, mentioned by almost everyone interviewed. In one municipality, this collective tale was referred to as “our tale of success” by all the respondents.

### Ethical considerations

Informed consent was first obtained from each head manager of care and social services in the municipalities, and information of the study was administered to the various care facilities. Verbal informed consent was then obtained from the participants who volunteered for the interviews. Participants were allowed to read a summary of their interview; three interviewees chose to read it, and had no objections. Participants were informed that the interviews would not contain any sensitive personal data, their identities and the collected data would be kept confidential and that they had the opportunity to withdraw from the study at any time. The study followed the Swedish research council’s [27] ethical guidelines.

## Findings

The presentation of the findings begins with the chronological story of the integrated care projects in the municipalities, followed by an exploration of the characters in the stories, using Gabriel’s framework of characterisations of stories as an interpretative tool. Next, self-presentations of a collective identity and conflicting stories follow.

### The story of success – from chaos to successful collaboration in integrated care

In all three municipalities, the story began with the description of a chaotic situation in the health and social care sector in Sweden in the 1990s. The implementation of the Community Care Reform in 1992 resulted in extensive changes in the counties, and the municipalities were awarded the main responsibility of caring for older people. In terms of narrative structure, the original state of affairs was depicted as an almost apocalyptic vision of a care system on the verge of collapse and the critique towards both health and social care was said to be harsh:

“There was large overcrowding in the ‘90s, in 1996, we had eight to nine patients in line in the corridors. Many older people were readmissions with fractures. We did have a lot of municipal faults, we had no knowledge about the chain of care, nobody knew what the other one was doing.” (Coordinator A)

At this point in time, the chain of care was neither spoken of nor implemented in the care of older people. The manager of social services in municipality C had been working long before the 1992 introduction of the Community Care Reform, and she contended:

“Nobody is perfect. We did get a lot of criticism from the County Administrative Board [inspectorate authority at the time].” (Social Services Manager C)

Out of the chaos emerged a need for a strong moral leadership and novel ideas, and the protagonists were there to deliver it, a few managers either took command or were given clear permission to reform the care system.

“We realised that nothing worked if we didn’t collaborate with the municipality […] Then we got in touch with the head manager in the municipality who also had a very difficult task with the budget, saving money, cutting down beds in the nursing homes. We were supposed to collaborate and we were just as upset all three of us, and that’s when we finally said, ‘We don’t give a damn about the money, what do we need?’ A few acute beds for the clinic and more beds in the municipality. We were in the same situation with a shortage of beds. Then we walked up to the county director and said, ‘We don’t want this fuss anymore. We want to organise this in another way’.” (Primary Care Manager B)

The meeting with the county director eventually resulted in the interim-ward seen today. However, the battle was not easily won. Complications had to be overcome; a struggle concerning resources between county and municipal managers finally ended with an agreement and a joint effort to work together in a novel way. The turning point occurred when the two managers of primary healthcare banded together with the manager in the municipality, forming a sort of troika. A similar storyline is evident in the two other municipalities, with complications resulting from disagreements and frustrations before they came to agree upon a common strategy. In all three municipalities, there were at least two managers (both male and female) leading the change process; the director of the hospital and one coordinator (A), two primary care managers and the head manager of social services (B) and the primary care physician and home healthcare manager (C).

Still, the crisis also held potential for the protagonists to mould the organisation in their own ways.

“It was such a mess […] But the crisis gave us the opportunity to test different things. And the municipality was in a state of collapse, too, when they realised they didn’t have money to [send to] all those nursing homes. And what were we supposed to do? We gathered all [our] staff on a regular basis and told them, ‘This is how we do it’. Nobody questioned what we did, and that’s not usually the case, is it?” (Primary Care Manager B)

The health and social care seemed to have reached the bottom line and with that came freedom for the managers to do what they deemed necessary. Several of the respondents involved in the changes at the time expressed rather considerate margins of discretion,

“We have a strong political governing and we get explicit objectives and they [the politicians] don’t interfere too much. We are the experts, together with the service user.” (Nursing Home Manager C)

The governing authorities were depicted as either acting with direction and clarity or not interfering, thus leaving a great deal of individual autonomy for the managers to decide what was best.

The overarching story of how collaboration between health and social care started was similar throughout the municipalities and resembled a classical epic-comic story plot [10], where a transition from an original and deficient state of affairs to a desirable situation was at hand, with the accomplishment of success. This was expressed as prizes won for best care and a good reputation, leading to easier recruitment of professionals, spreading strategies for integrated care and work procedures using research reports, evaluations and conferences and being recognised for good care in other municipalities and in different networks of care. Success was also defined in measurements such as short waiting times for older persons in hospital ready for discharge.

### Characters in the stories – heroes and victims

Not only is the plot similar in the stories told by various staff in the municipalities but so too is the heroic character descriptions of the leaders:

“We can’t overstate the importance of [x] as a carrier of the culture, she has been outstanding, to have the stamina to hold onto the project. She has personified Esther and she has done that very well.” (Social Services Manager A)

The very reason for not having any recruitment problems for registered nurses in the home healthcare system, unlike in other municipalities, was also said to be because of one of the main leaders of the change process:

“We don’t have difficulties in recruiting nurses, and I’d say that’s because of [x]. She’s famous, she’s a big name in this business, if I may say so.” (Social Services Manager C)

The leaders were attributed as heroes; engaged and charismatic, they saved the organisations from breakdown and their heroic accomplishments were connected to the service users’ welfare. Protagonists in epic-comic stories sometimes take on the role of a trickster, where a crisis is resolved through cunning and wit [10]. The hero is not solely courageous and dedicated but also different:

“The idea [of a general change] came from the county director, ‘We really need to do something different.’ And he was looking for some moderately deviant persons for this project and then I and a friend of mine were recruited […] the crisis was so severe, we could do whatever. Indeed, we would fix it and I thought that’s ok, I still live in [another area]. [laughter]” (Primary Care Manager B)

The manager presented himself as courageous but also different and unconventional, and if he failed in his mission he joked about not having to move because he already lived in another municipality. The leaders’ transformation of the organisations provides the unity of action, and all other activities and characters appear to be illuminated by their powers, unconventionality and almost eccentric qualities.

The characteristics of the protagonists also correspond with epic-comic stories; heroes with humour accompanied by assistants, sometimes described as accomplices [10]. Many meetings in different constellations were described where the cunning main characters managed to tackle obstacles and achieve success. The coordinator in municipality C accentuated the necessity of discussing needs in the first place and to hold back questions of budgets until later, as did the managers in municipality B, and she also stressed the need for perseverance:

“The politicians were not interested [at the] start, you have to persevere and infiltrate everywhere.” (Coordinator C)

The politicians appeared like an enemy-line that needed to be won over by edging in slowly and consistently. The change process was not described as being easily implemented in any of the municipalities, but rather as the result of a few individuals’ insistent and dedicated efforts.

In comparison, the characterisations of older people in need of care were outlined in terms of them being the victims waiting to be saved or as anonymous roles of extras. The heroes (managers) were talked about as individuals but older people were mostly talked about as characters on a group level: patients, service users and citizens, and in what respect the organising of care was most beneficial for them. However, the one exception was municipality A; they used the persona Esther when talking about the older person, which changed the character of the older person in their accounts. One of the advantages with their strategy was expressed as:

“Those older people who were not listened to before made their voices heard and became strong actors in this process.” (Coordinator A)

This was a statement which was part of the story of the Esther-project and was repeated by some of the respondents, albeit not all. How much agency older people had in the municipality was questioned by staff closer to the service user.

### The spirit of success forming a collective identity

In all of the interviews, a successful collaboration for integrated care was connected to self-presentations where a daring spirit, courage and novelty lay at the heart of the accounts. This was attributed to the leaders, but also to the organisations as a collective: “We are on the ball” (A) and “We are quick to seize upon” different challenges (A, B, C). Similarly, “It’s very significant for our network, we’re not afraid of trying” (Coordinator A). A permissive attitude and a trial and error approach was said to prevail in the municipalities and was explained as reasons for their success,

“It’s really unrestrained, no walls, no prestige. If you have a question, it’s okay to ask. It’s appreciated. Everyone realise[s] that we’re here for the patient and there’s no wins for me if the hospital is not working properly […] We’re one another’s success factor.” (Social Services Manager A)

The success was depicted as a win-win situation for both the service users and the care organisations. As a matter of fact, the whole municipality seemed to be included in this spirit of enthusiasm for trying out novel ideas. It was expressed as a certain spirit pertaining to the specific municipality, “This is how we are here in X”. Such claims were repeated in many of the interviews.

These stories are partial, told from the perspective of the involved parties, but in all interviews there were referrals to a collective “we”, disclosing a discursive collective identity, including a specific “spirit”, a daring and courageous attitude towards change and novel ideas embracing the whole community. The flexibility of everybody involved in the care and the individual autonomy and courage of many of the professionals in the diverse organisations were highlighted in these stories, leading to a shared sense of a successful collaboration and integrated care system where it was claimed the service user was always in focus.

### Conflicting stories

The story of success dominated throughout the interviews; however, there were small stories of a conflicting nature, and these were told as examples of situations where things did not work out well for either the staff or the service user. These stories were mostly told by staff working closer to the service user and included criticism of the collaborating organisations. There were complaints of the hospital being short-sighted and for pushing patients over to the short-term unit:

“When the chief [hospital] nurse called me and commanded me to find beds or they’d have to cancel surgery – that’s quite far from team work I’d say, it’s more like a threat. They shut down wards as soon as there’s a shortage of staff and push it over to us instead.” (Short-Term Unit Manager, Elderly Care A).

The nurse aide working in the same ward continued re-telling an incident from the previous summer:

“It was a huge crisis. Consequently, when poor ‘Esther’ came home and nothing worked we had to send her back again, and that’s a discussion between the two main responsible caregivers and ‘Esther’ shouldn’t really have to be in the middle of that.” (Nurse Aide A)

The position of the older person was used to justify the respondents’ claims of doing right, be it success or failure. The main goal and the accomplishments of professionals and staff were either said to be a benefit for the older person, or in cases of collaboration failures the service users were portrayed as victims, they were “falling between the cracks”.

Although older people’s benefit was emphasised as the main goal of the collaboration, their knowledge of the strategies for integrated care was questioned in municipality A:

“No, the service users and their next of kin don’t know about the Esther project. I can see that there’s a vision, but most people don’t even know there’s caregiver support. There’s so much information today, when you’re 80, 90 years old and there are so many concepts – Esther? Coaches? What is a ‘coach’? I mean the English term [sigh] I don’t know what we can do to get the message out.” (Caregiver Support A).

The conflicting stories in municipality A pointed towards a decrease in the support for the network model (Esther) from staff closer to the service user but remained a success story from the main managers’ point of view.

## Discussion

The subject for this analysis was storytelling and the function of stories as bridging cultural diversity in collaborations in health and social care in integrated care for older people in three Swedish municipalities. Each municipality had a main strategy to integrated care: a) a network model (the “Esther-model”), where the participating organisations collaborated on a voluntary basis without a hierarchical structure, b) pooling resources; a joint budget and management of one interim ward for patients and service users, and c) multi-professional teams. In the three municipalities, there was evidence of several factors identified as facilitating collaboration such as co-location, support from local politicians, low staff turnover and only one or two primary care units. This is in line with research on collaboration [2,4] as was the barrier of incompatible it-systems. In the theory of collaborative advantage, one way of managing cultural frictions is through balancing between autonomy and accountability [8]. In the stories, the individual autonomy of the managers in the care organisations was pronounced, but questions of accountability towards their own organisations were toned down, in favour of the joint integrated strategy. The result points at how cultural frictions may be overcome through the efforts put into a joint programme, where prominent leaders manage to rhetorically invoke a win-win situation for everybody involved, not the least for older people with complex needs. The content of the integrated care strategy seemed less important than the rhetorical power of influential managers.

The epic-comic type of story identified in the three municipalities seems particularly well suited to denote success as it was accomplished through characterisation of heroes’ (managers) and their improvising and discretionary work towards common goals; an accomplishment that was leading to collaborative advantage [8]. The storyline followed how a few managers had managed to turn a chaotic situation in the care of older people into successful collaborations to achieve integrated care where the older person always remained in focus. Although, the story was complicated with disagreements that had to be overcome, sometimes through wit and cunning, the plot was rather straightforward. In that way, the stories functioned as a means of interpreting and understanding complex events. The presence of a plot conveys temporal coherence, and even a minimal plot is enough to make sense of a story [12]. In addition, each hero had their ‘sidekick’, the leaders of the integrated care strategies worked in pairs and the ‘villains’ were understood as structures or specific individuals obstructing collaboration and integration of care, for example pushing their workload on other organisations. Inter-organisational collaboration is often disorganised and complicated. Thus, storytelling is crucial for sense-making in collaborating organisations because it guides action, delineates causality and conveys shared values and meaning [15].

In success stories there may be a strong tendency for key leaders to describe the outcome as a success and not as a failure [19]. There are several reasons as to why successes rather than failures are depicted, including the importance of keeping particular stakeholders pleased, political reasons and financial rewards, individual career advancement and the protection of self-esteem [19]. Moreover, success is more often attributed to the actions of the speaker and failures to external agents or causes. This leads to various stories of success and failures in different settings by different speakers. Conflicting stories of failures rather than success were rarely developed by the respondents, and they were mostly told by staff closer to the service user and not on a managerial level. Furthermore, the characterisations suggest a rationalisation of personal and other members’ actions. For example, portraying yourself or a colleague as a hero can be regarded as a way to reinforce the struggle to change and persevere in times of resistance and strain. The audience can associate with battles fought and won for the embodiment of the “true spirit of collaboration” [18, p. 66].

This analysis focused on one type of story which was similar in three municipalities using three different integrated care strategies. The epic-comic type of story was pivotal for the interviewees’ elaboration of the collective identity as expressed in their presentations of the collaborative experience. It was the most conspicuous and coherent story, and it was shared by almost all respondents contributing to a sense of belonging to one municipality where the hero’s spirit of novelty and courage was fundamental in uniting the diverse collaborating organisations. Nonetheless, stories serve the interests of an individual’s perceived interests [17]. Although collective identities are not static but changing, some stories may endure for long periods. One reason may be that they are systematically re-told to new members in the organisations [17]. However, there are many voices in organisations, and any identity claim acquires meaning in relation to “a network of others” [17, p. 743]. Other stories with different heroes and villains, based on the experiences of staff working closer to the care recipients were not voiced in this study. Although everyone has a voice, some are louder and more powerful than others [11]. Still, the managers’ stories of the successful collaboration in the integrated care project evoked pride and amusement in most respondents across the different care organisations. In this way, storytelling in collaboration is perhaps one way of overcoming cultural friction between different collaborating actors and can contribute to a coherent sense of what may be desirable to achieve together.

## Limitations

This paper provides an analysis of stories about successful collaboration by professionals working within the care sector in Sweden. Their approaches to integrated care were deemed successful in evaluations and in a benchmarking system; however, views from older people still have to be investigated. This study does not include any accounts or stories from service users or patients, and what is regarded as successful collaboration depends on who tells the story. Although different levels of staff were interviewed, the stories told by managers were more pronounced due to them being part of the collaborative process from the start. Future research on stories in collaborative experiences need to consider other perspectives than the managerial level.

## Conclusion

This study presents a narrative analysis of the stories of collaborative experience in three different approaches to integrated care for older people in Sweden. Stories were regarded as a vehicle to construct shared meaning and a collective identity [17]. The most significant finding was that a similar type of success story was evident in all three municipalities. The story was identified as an epic-comic type story where the transition from chaos to stability, or from failure to success, was lined with complications alongside a comical twist, with both obstructing and supporting characters, concluding in a happy ending; that is, successful collaboration leading to integrated care. Stories are sense-making devices and it is suggested that the success stories, and the storytelling, functioned as a means to bridge the diversities of different organisations involved in the collaboration, thus creating a collective identity beneficial for integrated care.
